# Feature Selection in Classification of Eye Movements Using Electrooculography for Activity Recognition

**DOI:** 10.1155/2014/713818

**Published:** 2014-12-09

**Authors:** S. Mala, K. Latha

**Affiliations:** Department of Computer Science and Engineering, Anna University, BIT Campus, Tiruchirappalli, Tamil Nadu 620 024, India

## Abstract

Activity recognition is needed in different requisition, for example, reconnaissance system, patient monitoring, and human-computer interfaces. Feature selection plays an important role in activity recognition, data mining, and machine learning. In selecting subset of features, an efficient evolutionary algorithm Differential Evolution (DE), a very efficient optimizer, is used for finding informative features from eye movements using electrooculography (EOG). Many researchers use EOG signals in human-computer interactions with various computational intelligence methods to analyze eye movements. The proposed system involves analysis of EOG signals using clearness based features, minimum redundancy maximum relevance features, and Differential Evolution based features. This work concentrates more on the feature selection algorithm based on DE in order to improve the classification for faultless activity recognition.

## 1. Introduction

Many researches in activity recognition and computer vision adopt gesture recognition as forefront [1]. Gesture recognition is an ideal example of multidisciplinary research; gestures convey meaningful information to interact with environment through body motions involving physical movements of fingers, arms, head, face, or body. There are many tools for gesture recognition like statistical modelling, computer vision, pattern recognition, image processing, and so forth. Feature extraction and feature selection are successfully used for many gesture recognition systems. Importance of gesture recognition laid in human-computer interaction applications from medical rehabilitation to virtual reality. Mitra and Acharya provided a survey on gesture recognition, particularly on hand gesture and facial expressions [2].

The goal of activity recognition is to provide information that allows a system to best assist the user with his or her task. Activity recognition has become an important area for a broad range of applications such as patient monitoring, vigilance system, and human-computer interaction. Bulling is the first to describe and apply eye base activity recognition to the problem of recognition of everyday activities [3]. Eye movements have plentiful information for activity recognition.

Bulling also described and evaluated algorithms for detecting 3 eye movement characteristics of EOG signals, saccades, fixations, blinks [4]. One of the eye movement characters, blink, plays major role in activity recognition. Blink serves as the first line of ocular protection. The wiping action of the lid can remove dust, pollens [5]. According to Ousler et al. blink rate can indeed serve as an indicator of the cognitive or emotional state of the blinker [6]. Cummins states blinking might also contribute to social interaction by strengthening mutual synchronization in movements and gestures [7]. Leal & Vrij represented blinks in increased cognitive demand during lying [8]. Ponder and Kennedy reported about blinks during emotional excitement [9] and Hirokawa depicted more frequent blinks in highest nervousness [10]. Cognitive processes have a substantial impact on blink rates, mentally taxing activities like memorization or mathematical computation being associated with an increase in blink rate and inattentiveness (daydreaming) and stimulus tracking being associated with low blink rates. Blinks together with other eye movements would be possible sources of useful information in making inference about people's mental states.

One of the possibilities to detect eye movements is EOG, which is a technique for measuring the resting potential of the retina [11, 12]. The following lists some of the wide range of applications of EOG.

- Wijesoma et al. used EOG for guiding and controlling a wheelchair for disabled people [13].
- Usakli and Gurkan used EOG for using virtual keyboard [14].
- Deng et al. used EOG for operating a TV remote control and for a game [15].
- Talaba used EOG for visual navigation metaphor [16].
- Gandhi et al. used EOG for controlling multitask gadget [17].
- Bulling et al. used EOG for activity recognition [18].

Feature selection is important and necessary when a real world application has to deal with training data of high dimensionality. There are many features selection approaches available in the literature. Some of the hybrid approaches are listed as follows.

- García-Nieto et al. presented a Differential Evolution based approach for efficient automated gene subset selection using DLBCL Lymphoma and Colon tumor gene expression datasets. The selected subsets are evaluated by means of the SVM classifier [19].
- Li employed a DE-SVM model that hybridized DE & SVM to improve the classification accuracy of road icing forecasting using feature selection [20].
- Xu and Suzuki proposed a feature selection method based on sequential forward floating selection to improve performance of a classifier in the computerized detection of polyps in CT colonography (CTC). In this work feature selection is coupled with SVM classifier and maximized the area under the receiver operating characteristic curve [21].
- Kuo et al. proposed kernel based feature selection method to improve the classification performance of SVM using hyperspectral image datasets [22].
- Güven and Kara employed artificial neural network analysis of EOG signals for the purpose of distinguishing between subnormal and normal eye [23]. This enables the physician to make a quick judgment about the existence of eye disease with more confidence. Only binary classification of EOG signal has been performed in this work.

From the literature review it can be observed that there are a number of EOG applications being developed. Still necessary feature selection algorithms need to be developed, evaluated, and used to produce substantial improvements in communications with disabled people by using eye movements and to make inference about person's cognitive state.

This paper presents a hybrid feature selection technique based on DE for activity recognition using eye movements by EOG signals which can identify a subset of most informative, eye movement characteristics amongst all eye movement characteristics. This method is used as an optimizer before the classifier to EOG signal features for recognizing activities like read, browse, write, video, and copy. The benefits of the feature selection approach include improving the efficiency of activity recognition since only a subset of eye movement is used and assisting SVM to attain satisfied accuracy.

## 2. Methodology

Here we explain the preprocessing, feature selection (with CBFS, mRMR, and DEFS), SVM classifier, and the model evaluation strategy used in this work.

### 2.1. Dataset Description: Recognition-of-Office-Activities

The EOG data used in this study are collected from the Andreas Bulling's “recognition-of-office-activities” dataset (https://www.andreas-bulling.de/datasets/recognition-of-office-activities/). Eight participants took part during this study. For about 30 minutes the participants were involved in two continuous activity sequences. The total dataset is about eight hours. The data aggregation of this work is grounded on office based activities performed in random order paper reading, taking notes, watching video, and net browsing. Additionally, the dataset includes a period of rest (the NULL class). These activities are all generally performed during a usual working day.

These experiments were carried out in a well-lit workplace during normal working hours. Participants were seated ahead of 2 seventeen inch flat screens with a resolution of 1280 × 1024 pixels on which a video player, a browser, and text for copying in a word processor were on-screen. Sheets of paper and a pen were presented on the table close to the participants for reading and writing tasks. No constraints were forced with type of website and manner of interaction of browsing task.

EOG signals were picked up using an array of five 24 mm Ag/AgCl wet electrodes from Tyco Healthcare placed around the right eye. The horizontal signal and vertical signal were collected using two electrodes for each and the fifth electrode was placed on the forehead for the signal reference. EOG data is captured employing a commercial EOG device known as TMSI (Twente Medical Systems International) Mobi8 that integrates instrument amplifiers with 24 bit ADCs. Mobi8 tends to have better signal quality. Mobi8 was worn on a belt around each participant's waist and recorded four channels EOG at a sampling rate of 128 Hz. The behaviours of participants in specific phases (read, browse, write, video, copy, and rest) were observed by an annotated activity changes with wireless remote control and their nature in daily life during regular working hours. Context Recognition Network Toolbox (CRNT) was used for handling data recording and synchronization.

### 2.2. Baseline Drifts Removal and Filtering EOG Signals

For preprocessing, this work adapts median filter and baseline drift removal 1D wavelet decomposition at level nine using Daubechies wavelets on the signal component [18].

**Figure 1:**
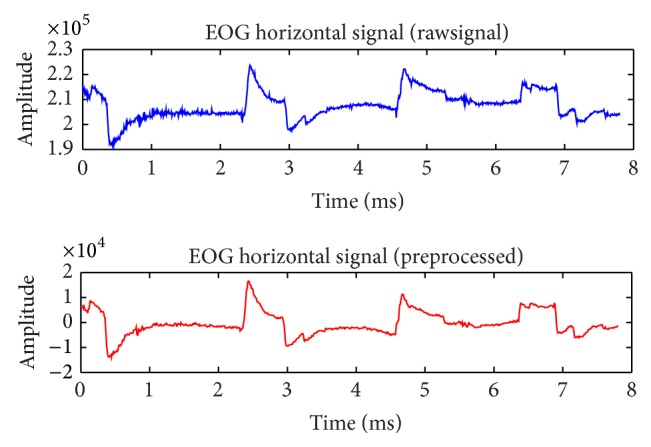
Before and after preprocessing (EOG) signals.

Figure 1 shows EOG signals of horizontal, before and after baseline drifts removal and filtering.

Table 1 lists corresponding mean square error (MSE) and peak signal to noise ratio (PSNR) for two participants with thousands of samples.

### 2.3. Basic Eye Movement Type Detection

Various activities using eye movements by EOG can be portrayed as a regular pattern by a specific sequence of saccades and short fixations of similar duration. The amplitude change in signals varies for various activities which can be used in identifying the regular office activities. The reading activity using EOG is patterned by small saccades and fixations. No large change in amplitude is included in reading. This is due to small eye movement between the words and fast eye movement between ends of previous line and beginning of next line. Writing was similar to reading, yet it required greater fixation duration and greater variance. It was best described using average fixation duration. Copying activity includes normal back and forth eye movements which involves saccades between screens. This was reflected in the selection of small and large horizontal saccade features, as well as variance in horizontal EOG fixations. In contrast, watching a video and browsing are highly unstructured. These activities depend on the video or website being viewed. These results propose that, for tasks that involve a known set of specific activity classes, recognition can be streamlined by only choosing eye movement features known to best describe these classes.

The steps in basic eye movement type detection for EOG based action recognition are (1) noise and baseline drift removal, (2) basic eye movement detection, and (3) feature extraction. The basic eye movements' interpretation and detections are carried out using wavelet coefficients.

Eye movement characteristics like saccades, fixations, and blink patterns are separated from EOG signals using continuous wavelet transform (CWT) 1D wavelet coefficients using a Haar mother wavelet at scale 20 [18]. The preprocessed EOG signal components (EOGh, EOGv-denoised, baseline drift removed) are given as input to CWT:

(1) $$\begin{array}{c} C_{a}^{b}=\int_{R}s\left(t\right)\frac{1}{\sqrt{a}}\bar{\psi \left(\frac{t-b}{a}\right)}dt. \end{array}$$

The wavelet coefficient (*C*_*a*_^*b*^) of signal (*s*) at scale *a* and position *b* is defined by (1):

(2) $$\begin{array}{c} N_{i}=\left\{\begin{array}{cc} 1, & \forall i\text{:}\;C_{i}\left(s\right)<-{\text{th}}_{\mathrm{sd}}\\ -1, & \forall i\text{:}\;C_{i}\left(s\right)>{\text{th}}_{\mathrm{sd}},\\ 0, & \forall i\text{:}\;-{\text{th}}_{\mathrm{sd}}\leq C_{i}\left(s\right)\leq {\text{th}}_{\mathrm{sd}}. \end{array}\right. \end{array}$$

By applying a threshold th_*sd*⁡_ = 2000 on the coefficients *C*_*i*_(*s*) = *C*_*i*_^20^(*s*), CWT creates a vector *N* with elements *N*_*i*_ based on (2). This step divides EOGh (horizontal EOG) and EOGv (vertical EOG) in saccadic (*N* = 1, −1) and nonsaccadic (fixation) segment (*N* = 0).

We used the same wavelet coefficients to detect blinks in EOGv. Features related to the eye movements using EOG signal were calculated separately for two EOG (horizontal and vertical) signals for each participant. Statistical features such as mean, variance, and maximum value, minimum value based on saccades, fixations, and blinks are extracted from this work. Different characteristics of EOG signals result in different changes in the coefficients. Totally 210 statistical features are extracted from this work.

**Figure 2:**
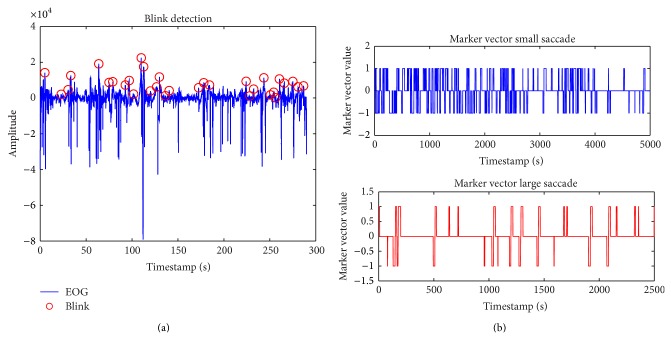
(a) Blink detection from EOGv. (b) Saccades from EOGh.

Eye movement characteristics such as saccades and blink patterns of EOG (horizontal and vertical) signals are shown in Figure 2.

### 2.4. Feature Selection

#### 2.4.1. Minimum Redundancy Maximum Relevance (mRMR)

The minimum redundancy maximum relevance (mRMR) [24, 25] algorithm is a sequential forward selection algorithm. It uses mutual information to analyze relevance and redundancy. The mRMR scheme selects the features that correlate the strongest with a classification characteristic and combined with selection features that are mutually different from each other having high correlation and it is denoted by the following equation:

(3) $$\begin{array}{c} J\left(X_{n}\right)=I\left(X_{n};Y\right)-\frac{1}{\left|S\right|}\sum_{X_{i}\in S}I\left(X_{n};X_{i}\right), \end{array}$$

where *I*(*X*_*n*_; *Y*) is the measure of dependence between feature *X*_*n*_ and objective *Y*. $J=I(X_{1:n};Y)-I(X_{\begin{array}{c} 1:n-1 \end{array}};Y)$ is the difference in information with and without *X*_*n*_.* S* is the feature set and |*S*| is the number of features.

#### 2.4.2. Clearness Based Feature Selection (CBFS)

CBFS [26] calculates the distance between the objective sample and the center of every class, and then compares the class of the nearest center with the class of the objective sample. The similarity ratio of all samples in a feature becomes a clearness value for the feature [26].

Clearness based feature selection (CBFS) algorithm is a type of filter method. Clearness means the detachment between classes in a feature. If (clearness of feature *f*_2_) > (clearness of feature *f*_1_), then *f*_2_ is more useful for classification than *f*_1_.

**Step 1 .**

The centrist for read and write is calculated by average operation. This is the median point of a class. Med(*f*_*i*_, *j*) represents the median point of class *j* in the feature *f*_*i*_, and it is calculated by the following equation:

(4) $$\begin{array}{c} \text{Med}\left(f_{i},j\right)=\frac{1}{k}\overset{k}{\underset{r=1}{\sum }}\left(Xri\in \text{class}\;\;j\right), \end{array}$$

where *k* is a number of samples of class *j*.

**Step 2 .**

For each *x*_*ij*_, the sample predicted class is calculated. After calculating the distance between *x*_*ij*_ and Med(*f*_*j*_, *c*_*i*_) for all classes, this work takes the nearest centrist Med(*f*_*j*_, *s*) and *s* is a predicted class label for *x*_*ij*_. The distance between *x*_*ij*_ and Med(*f*_*j*_, *t*) is calculated by the following equation:

(5) $$\begin{array}{c} D\left(x_{ij},\text{Med}\left(f_{j},t\right)\right)=\left|x_{ij}-\text{Med}\left(f_{j},t\right)\right|. \end{array}$$

**Step 3 .**

Calculate *n* × *m* matrix *M*_2_. This matrix contains a matching result of predicted class label and correct class label in *C*_Score_. *M*_2_(*i*, *j*) is calculated by

(6) $$\begin{array}{c} M_{2}\left(i,j\right)=\left\{\begin{array}{cc} 1 & \text{if}\;M_{1}\left(i,j\right)=C_{i}\\ 0 & \text{if}\;M_{1}\left(i,j\right)\neq C_{i}. \end{array}\right. \end{array}$$

**Step 4 .**

Calculate *C*_Score_ (*f*_*i*_). Initially we calculated *C*_Score_ (*f*_*i*_) by

(7) $$\begin{array}{c} C_{\text{Score}}\left(f_{i}\right)=\frac{1}{n}\overset{n}{\underset{r=1}{\sum }}M_{2}\left(r,i\right). \end{array}$$

The range of *C*_Score_(*f*_*i*_) is [0, 1]. If *C*_Score_ (*f*_*i*_) is close to 1, then it indicates that classes in feature *f*_*i*_ are grouped well and elements in *f*_*i*_ can be clearly classified.

### 2.5. Proposed Method

The proposed method first identifies essential features by applying a threshold (th_*c*_) in correlated values among features. This stage reduces the feature space dimensionality. If correlation (*c*) between the features satisfies the threshold (th_*c*_ < 0.8) those features are selected for the next stage. After removing tautological features approximately 184 features are selected for this work.

The reduced feature space is given to the DEFS algorithm for detecting the significant feature subset through machine learning algorithm such as maximum a posterior approach. Here for the projection of features into feature subspace we go for kernelized Bayesian structure. This method is used as an optimizer before the classifier to EOG signal features for recognizing activities like read, browse, write, video, and copy.

**Figure 3:**
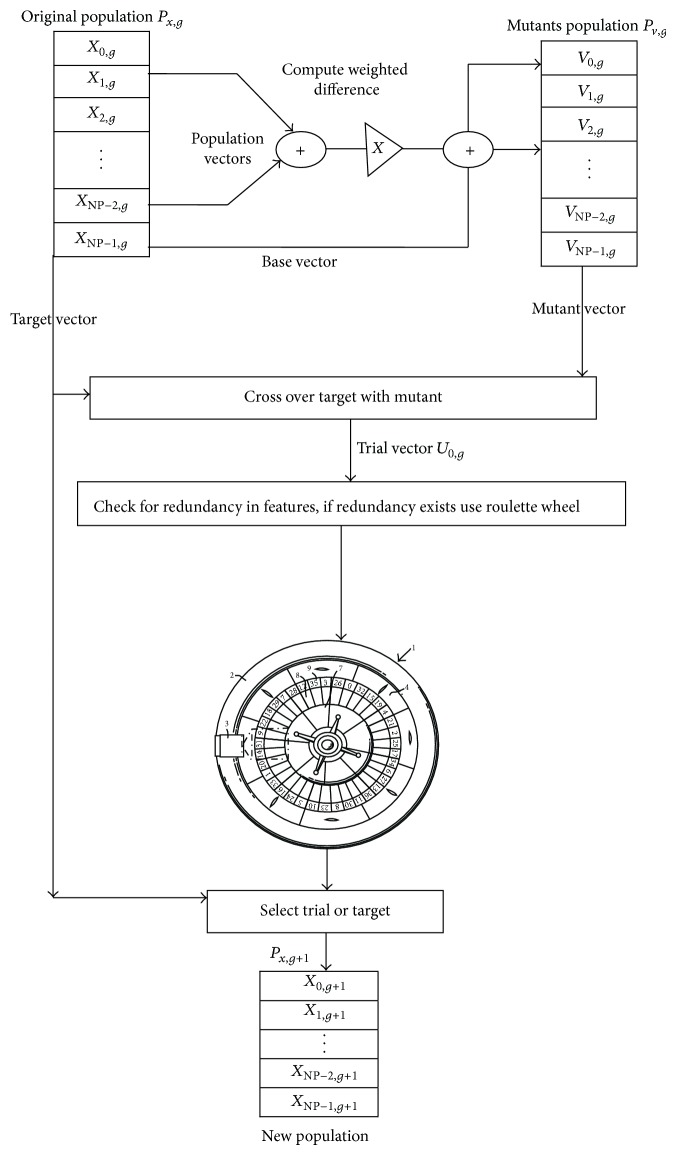
Differential Evolution operations.

Figure 3 illustrates the steps involved in the Differential Evolution process [27]. The first step in the DE optimization method is to generate a population (NP × D) of NP members, each of D-dimensional real-valued parameters. In order to improve the DE subset selection efficiency, we are going to change the fitness function in terms of maximum a posteriori probability. The kernel distribution is appropriate for EOG features since it has a continuous distribution. The EOG features may be skewed and have multiple peaks; we can use a kernel which does not require a strong assumption. The kernel needs additional time and memory for computing than the normal distribution. For every feature we model with a kernel the Naive Bayes classifier or every category based on the training data for that class. The default kernel is normal, and also the classifier selects a width automatically for every class and feature. The stopping criterion was defined as reaching the maximum number of iterations. The chosen fitness function was the classification error rates achieved by the Naive Bayes classifier with kernel distribution.

In this work parameter *F* is assigned dynamically, CR with the value of 0.8, the number of dimensions of the problem (#features) D is 15, the population used is 50, and the number of generations is 10:

(8) vj,i.g=xj,r0.g+F×xj,r1.g−xj,r2.g,wherei=0  to  NP−1j=1  to  D−1xr1,xr2=two  population  membersF∈0,1  scale  factor  g  is  generation.

For each target vector mutant vector is created by using (8):

(9) uj,i.g=vj,i.gif  rand(0,1)≤Crxj,i.gotherwisewhereui+j  is  jth  dimension  from  ith  trial  vectorg=populationCr=Crossover  probability.

The parent vector is mixed with the mutated vector to produce a trial vector as in (9).

DE employs uniform crossover. Newly generated vector results in a lower objective function value (fitness). The randomly chosen initial population matrix of size (NP × DNF) containing NP randomly chosen vectors *i* = (0,1,…, NP − 1) is created. DNF is desired number of features to be selected. We made search limited by the total number of features (NF = 15). Individual lower boundary of the search space is *l* = 1 and upper boundary is H = NF = 15. Each new vector from initial population is indexed as 0 to NP − 1. The steps in the DE process are as follows: the difference between two population numbers (P1, P2) is added to a third population member (P3). The result (R4) is subject to crossover with candidate for replacement (C5) to obtain a proposed (PR6). The proposed system is evaluated and replaces the candidate if it is found to be the best. In our scheme the probability of each feature is calculated and used as weighting to replace the duplicated features. All the time the features are not in a linear manner. So we only go for nonlinear kernel structure to enclose the features in feature space. The objective of this work is reducing the complexities of evolutionary algorithm and improving the fitness validation by machine learning probabilistic kernel classifier by considering the nonlinearity of inputs.

Table 2 shows the number of samples taken for each activity, features identified from each activity, and the performance summary for each activity with and without DEFS features.

### 2.6. SVM

Activity classification involves a set of *m* samples labelled with a class. A sample is a vector *x*_*i*_ = [*x*_*i*1_, *x*_*i*2_,…, *x*_*in*_] of *n* features, where *x*_*ij*_ [*f*_*j*min⁡_, *f*_*j*max⁡_] is the activity recognition value for the* j*th feature of the *i*th sample (1 ≤ *i* ≤ *m*; 1 ≤ *j* ≤ *n*). The label associates a class value, class_*i* *C* with the sample *x*_*i*_, where *C* = {class1, class2,…, class*k*} is a set of class values for the samples. In this work, we consider SVM to classify samples with six possible class values (*k* = 6).

SVM handles nonlinear data by using a kernel function [28]. The kernel maps the data into a feature space. The nonlinear function is learned by a linear learning machine in a high dimensional feature space. This is known as kernel trick which implies that the kernel function transforms the data into a higher dimensional feature space to form feasibly linear separation [29]:

(10) $$\begin{array}{c} \text{Linear:}\;K\left(x_{i},x_{j}\right)=x_{i}^{T}x_{j}. \end{array}$$

The kernel function used in this work is linear kernel, meaning dot product which is shown in (10):

(11) $$\begin{array}{c} \omega \left(\alpha \right)=\overset{n}{\underset{i=1}{\sum }}\alpha_{i}-\frac{1}{2}\overset{n}{\underset{i=1,j=1}{\sum }}\alpha_{i}\alpha_{j}K\left(x_{i},x_{j}\right), \end{array}$$

(12) $$\begin{array}{c} \omega =\overset{n}{\underset{i=1}{\sum }}\alpha y_{i}x_{i}. \end{array}$$

There is a class of functions *K*(*x*, *y*) with a linear space *S* and a function *φ* mapping *x* to *S* such that *K*(*x*, *y*) = 〈*φ*(*x*), *φ*(*y*)〉. The dot product takes place in the space *S*. Solve (11) and get the *α*_*i*_, which maximize *ω*  using (12):

(13) $$\begin{array}{c} f\left(x_{\text{new}}\right)=\mathrm{sign}\left(\overset{n}{\underset{i=1}{\sum }}\alpha y_{i}K\left(x_{i,}x_{\text{new}}\right)+b\right). \end{array}$$

The classifier is defined by (13). We used 75% of each activity for training the classifier. The classifier performance is improved by our proposed hybrid feature selection method.

### 2.7. Performance Evaluation Criteria

We adopted the following criteria to evaluate the performance of the classifier. We used Mat lab to calculate the accuracy of the classifier:

(14a) $$\begin{array}{c} \text{Precision}=\frac{\text{TP}}{\text{TP}+\text{FP}}, \end{array}$$

(14b) $$\begin{array}{c} \text{Recall}=\frac{\text{TP}}{\text{TP}+\text{FN}}, \end{array}$$

(14c) $$\begin{array}{c} F\text{-Measure}=\frac{2\text{TP}}{2\text{TP}+\text{FP}+\text{FN}}, \end{array}$$

(14d) $$\begin{array}{c} \text{Accuracy}=\frac{\text{TP}+\text{TN}}{\text{TP}+\text{TN}+\text{FP}+\text{FN}}, \end{array}$$

where TP, TN, FP, FN represents true positive, true negative, false positive, and false negative.

The sensitivity, specificity, true positive rate (TPR), false negative rate (FPR), and accuracy (ACC) were calculated by using (14a)–(14d).

The detailed performance of the proposed approach with DEFS features is listed in Table 3. The precision for video activity is higher than other activities. The recall for writing activity is slightly higher than other two feature selection methods. The detailed performance of the clearness based features with SVM and minimum redundancy maximum relevance features with SVM approach is listed and is also compared with DEFS features. The result shows that the proposed approach best performs with the other two methods.

**Figure 4:**
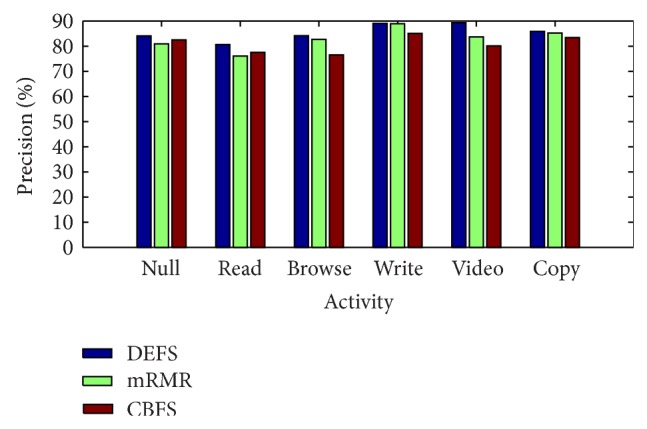
Precision for each activity by proposed DEFS based features with mRMR, CBFS features.

Figure 4 shows that precision for each activity is high for the proposed DEFS based feature selection compared to the other two feature selection methods, mRMR and CBFS. For write activity the proposed DEFS based feature selection and mRMR nearly provide the same results. For copy activity all the three feature selection methods provide almost the same precision values. For video activity, the proposed method outperforms well. Null and read activity precision shows that the CBFS features provide the best result when compared to mRMR features.

## 3. Results and Discussion

Feature extracted addresses the problem of finding a more informative set of features. The resultant feature subset shows that the most informative features in EOG signals are saccades and blinks. The statistical measures for EOG signal analysis proved to be very useful in searching the feature space by using hybrid feature selection based on differential evolution. The SVM with linear kernel is used for classification in EOG datasets before and after feature selection. The results of 10-fold cross-validation are listed in Table 3. In the table, we report performance by accuracy. The dataset is divided into training set with three-fourth of original and rest one-fourth of testing instances. We focus on Differential Evolution based feature selection algorithm in order to improve the classification. The detailed accuracy of class results is shown in Table 2 with an emphasis on the difference before and after feature selection. The results in Table 2 shows, class accuracy is increased with minimum number of features. The selected features improve the performance in terms of lower error rates and the evaluation of a feature subset becomes simpler than that of a full set. When the results are compared with CBFS and mRMR feature selection, the classifier (SVM) performance (ACC = 83.33%) is significantly improved with proposed features.

## 4. Conclusions

A hybrid feature selection method was employed in EOG signals based on the Differential Evolution and the proposed method is compared with CBFS and mRMR feature selection. The differential evolutionary algorithm is utilized to give powerful results in searching for subsets of features that best interact together through supervised learning approach. EOG dataset with high dimensionality and number of target classes (*N* = 6) were considered to test the performance of the proposed feature selection. What better fits our case is lower classification error rate, which is attained as 0.16. The classification performance is significantly improved when using the proposed feature selection algorithm that uses differential evolution based on a posteriori probability. This method followed by wavelet feature extraction presents powerful results in terms of accuracy (ACC = 83.33%) and an optimal subset of features with size (*S*) 15.

## Figures and Tables

**Table 1:** MSE and PSNR values.

Activity	Null	Read	Browse	Write	Video	Copy
Samples number	320315	290963	302385	317092	304170	303441
MSE						
Before filtering	4.1736*e* + 06	3.1071*e* + 06	7.550*e* + 05	2.1893*e* + 06	1.0267*e* + 06	3.1546*e* + 06
After filtering	**1.5957** *e * ** + 05**	**5.3059** *e * ** + 04**	**8.9856** *e * ** + 04**	**1.8710** *e * ** + 05**	** 8.4765** *e * ** + 04**	**1.1215** *e * ** + 05**
PSNR						
Before filtering	30.4026	29.5266	30.3008	31.0553	30.5343	31.0118
After filtering	**44.9982**	** 46.9136**	** 39.6886**	** 42.7312**	**42.2990**	**45.5411**

**Table 2:** Performance summary.

Performance	Null	Read	Browse	Write	Video	Copy	All
Samples number	320315	290963	302385	317092	304170	303441	1838366
Number of features	232	211	218	225	218	218	210
Accuracy (all features)	82%	67%	62%	73%	83%	68%	72.5%
Accuracy (with DEFS-15 features)	87%	77%	79%	84%	88%	85%	**83.33%**

**Table 3:** Detailed performance of the proposed approach with CBFS and mRMR features.

Activity	Features	TP (%)	FP (%)	Precision (%)	Recall (%)	*F*-Measure (%)
Null	DEFS	**75.31**	**14.20**	**84.14**	**92.24**	**87.99**
	mRMR	65.50	15.41	80.95	93.32	86.70
	CBFS	67.60	14.30	82.54	89.89	86.06

Read	DEFS	**71.60**	**17.12**	**80.70**	**93.74**	**86.73**
	mRMR	58.30	18.29	76.11	91.79	83.22
	CBFS	57.29	16.58	77.56	82.27	79.84

Browse	DEFS	**75.17**	**14.15**	**84.16**	**93.75**	**88.69**
	mRMR	68.98	14.38	82.75	95.17	88.53
	CBFS	54.00	16.50	76.60	81.82	79.12

Write	DEFS	**81.23**	**09.92**	**89.12**	**97.05**	**92.91**
	mRMR	79.10	08.85	88.94	94.56	92.19
	CBFS	69.24	12.10	85.12	90.39	87.68

Video	DEFS	**85.51**	**10.20**	**89.34**	**99.38**	**94.09**
	mRMR	67.41	13.09	83.74	90.23	86.86
	CBFS	62.37	15.44	80.16	86.16	83.05

Copy	DEFS	**80.21**	**13.18**	**85.89**	**97.61**	**91.37**
	mRMR	70.34	10.28	85.25	87.66	87.45
	CBFS	56.44	11.17	83.48	81.88	82.67
